# Development and Validation of a Chemostat Gut Model To Study Both Planktonic and Biofilm Modes of Growth of *Clostridium difficile* and Human Microbiota

**DOI:** 10.1371/journal.pone.0088396

**Published:** 2014-02-06

**Authors:** Grace S. Crowther, Caroline H. Chilton, Sharie L. Todhunter, Scott Nicholson, Jane Freeman, Simon D. Baines, Mark H. Wilcox

**Affiliations:** 1 Leeds Institute of Biomedical and Clinical Sciences, Faculty of Medicine and Health, University of Leeds, Leeds, United Kingdom; 2 Department of Microbiology, Leeds Teaching Hospitals NHS Trust, Leeds, United Kingdom; 3 School of Life and Medical Sciences, Department of Human and Environmental Sciences, University of Hertfordshire, Hatfield, United Kingdom; 4 School of Science, University of West Scotland, Hamilton, United Kingdom; Universidad Andres Bello, Chile

## Abstract

The human gastrointestinal tract harbours a complex microbial community which exist in planktonic and sessile form. The degree to which composition and function of faecal and mucosal microbiota differ remains unclear. We describe the development and characterisation of an *in vitro* human gut model, which can be used to facilitate the formation and longitudinal analysis of mature mixed species biofilms. This enables the investigation of the role of biofilms in *Clostridium difficile* infection (CDI). A well established and validated human gut model of simulated CDI was adapted to incorporate glass rods that create a solid-gaseous-liquid interface for biofilm formation. The continuous chemostat model was inoculated with a pooled human faecal emulsion and controlled to mimic colonic conditions *in vivo*. Planktonic and sessile bacterial populations were enumerated for up to 46 days. Biofilm consistently formed macroscopic structures on all glass rods over extended periods of time, providing a framework to sample and analyse biofilm structures independently. Whilst variation in biofilm biomass is evident between rods, populations of sessile bacterial groups (log_10_ cfu/g of biofilm) remain relatively consistent between rods at each sampling point. All bacterial groups enumerated within the planktonic communities were also present within biofilm structures. The planktonic mode of growth of *C. difficile* and gut microbiota closely reflected observations within the original gut model. However, distinct differences were observed in the behaviour of sessile and planktonic *C. difficile* populations, with *C. difficile* spores preferentially persisting within biofilm structures. The redesigned biofilm chemostat model has been validated for reproducible and consistent formation of mixed species intestinal biofilms. This model can be utilised for the analysis of sessile mixed species communities longitudinally, potentially providing information of the role of biofilms in CDI.

## Introduction

The human colon is a highly complex and diverse ecosystem lined by a mucous membrane. Bacterial populations within the gastrointestinal tract exist in two forms; planktonic free-floating communities within the lumen, and sessile bacteria within mucosal-associated biofilm communities. The mucus layer protects the underlying epithelium from the external environment, including pathogens [1]. The microorganisms present in the gastrointestinal tract often form multispecies sessile communities attached to the mucosal layer [2], [3]. Mucosal communities interact closely with host epithelial cells, and thereby may have a greater influence than planktonic populations on disease pathogenesis and immunomodulatory responses. Despite these potential important roles, many intestinal microbiota studies are derived from faecal samples and only provide information on planktonic populations. Mucosal populations are difficult to investigate in healthy individuals due to the physical inaccessibility of the gut; thus, there has been restricted study of these communities. The degree to which composition and function of faecal and mucosal microbiota differ remains unclear, although some reports suggest distinct differences in microbial composition at the two sites [3], [4]. The use of *in vitro* models of the gastrointestinal tract have been used to model planktonic and sessile communities [5]–[7], but typically over short time frames.

*C. difficile* infection (CDI) is a major threat in hospital and long-term care facilities worldwide and associated with significant financial burden [8]. Recurrence of disease symptoms is a particular challenge [9], [10]. Perturbation of the normal gut microflora (e.g. by antibiotic therapy) is the main risk factor for CDI [11], [12]. However, as the sessile intestinal microbiota populations are not well studied, the role of biofilms in CDI and recurrence is poorly understood. Biofilms have been implicated in chronic disease [13], and so may well be an important factor in recurrence of CDI.

In order to investigate long-term changes in intestinal microbiota, for example following antimicrobial therapy or in CDI development, more temporally robust models are required. We describe the development and characterisation of an *in vitro* continuous chemostat human gut model that facilitates the formation of mature mixed species biofilms, which can be longitudinally harvested and analysed separately. This chemostat system was validated for the consistent formation of 18 independent rod-associated biofilm structures, in both single- and triple-stage gut model systems.

## Methods

### *In vitro* human gut model

We have previously described the use of a triple-stage chemostat human gut model to study the interaction between antimicrobial agents, the indigenous gut microbiota and *C. difficile* [14]–[16]. This model has been validated against physicochemical and microbiological measurements from the intestinal contents of sudden death victims[17], and comprises three pH-maintained (pH 5.5±0.2, vessel 1; pH 6.2±0.2, vessel 2 and pH 6.8±0.2, vessel 3) fermentation vessels connected in a weir cascade formation. The model is sparged with nitrogen and top-fed with a complex growth medium at a controlled rate (D = 0.015 h^−1^). Constituents and preparation of growth medium for the gut model are as described previously [14]. The gut model was inoculated with a faecal emulsion (∼10% w/v in pre-reduced PBS) prepared from *C. difficile*-negative faeces of three healthy elderly (≥60 yrs) volunteers with no history of antimicrobial therapy for at least 3 months.

### Biofilm human gut model

This original model was adapted to facilitate the formation of macroscopic, mixed species biofilm by utilising a redesigned biofilm vessel. Eighteen glass rods were inserted into the vessel through the lid and positioned to ensure the sampling portion of the rod extended across the liquid/gas interface. Rods were screwed into the lid system and anaerobic atmosphere maintained by the delivery of nitrogen gas to the vessel via a circular pipe, with drilled holes interspersed on the surface to encourage even distribution of gas flow across the rods.

### Ethics Statement

The only ethical consideration necessary for the use of an *in vitro* gut model is for the collection and use of faecal donations from adult volunteers. This approval was provided from the Leeds Institute of Health Sciences and Leeds Institute of Genetics, Health and Therapeutics and Leeds Institute of Molecular Medicine, University of Leeds joint ethics committee (reference HSLTLM/12/061). Donors were provided with a participant information sheet, clearly outlining requirements and use of samples, and stating that by providing the sample to the researcher, participants were consenting for that sample to be used in research involving the *in vitro* gut model. To preserve anonymity of participants, no written consent was recorded. The need for written informed consent from the participants was waived by the University of Leeds joint ethics committee.

### Experimental design

#### Model A1 and A2

**Figure 1:**
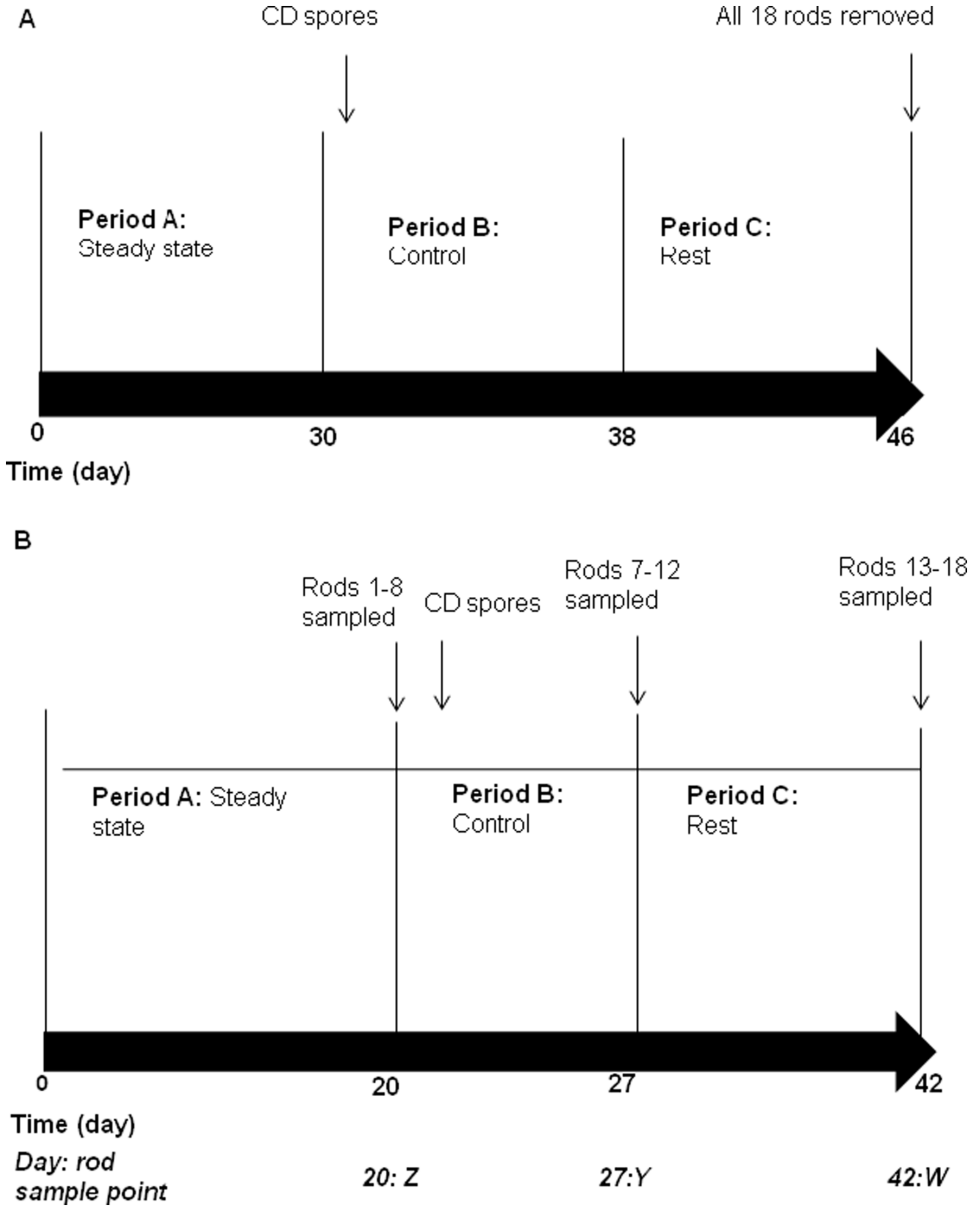
Experimental designs used in this study. Experimental design of (a) single stage (model A1 and A2) and (b) triple stage biofilm gut model validation experiments (models B1 and B2). Vertical line represents the last day of each time period. CD, *C. difficile* PCR ribotype 027 spores.

Duplicate single-stage gut model validation experiments (figure 1A).

The redesigned biofilm model was assembled as a single stage vessel only that was top fed with growth medium. Nine ground/roughened and nine smooth glass rods were incorporated. Pooled human faecal emulsion was added to the vessel, the media pump was started and the system was left without intervention to allow bacterial populations to equilibrate for 31 days (period A, figure1 A). On day 31 a single inoculum of *C. difficile* PCR ribotype 027 spores (∼10^7^ cfu) were added to the vessel (period B). The model was left without interventions until day 46 when all 18 rods were removed from the model for analysis. Planktonic indigenous gut microbiota (periods A–C), *C. difficile* total viable counts, spores and cytotoxin (periods B and C) were determined at regular intervals for the duration of the experiment. The experiment was performed twice to determine reproducibility of results.

#### Model B1 and B2

Duplicate triple-stage gut model validation experiments (figure 1B).

A more complex redesigned biofilm model comprising three vessels was assembled. Eighteen ground/roughened rods were inserted into the biofilm vessel, situated in vessel 3. Pooled human faecal emulsion was added to all three vessels, the media pump started, and the system was left without intervention for 19 days (figure 1B, period A). On day 20, rods 1–6 (sample point Z) were selected from all areas of the vessel, removed, and bacterial populations enumerated. A single inoculum of *C. difficile* PCR ribotype 027 spores (∼10^7^ cfu) were added to vessel 1 of the model on day 21 and the system was left without further interventions until rods 7–12 (sample point Y) were sampled on day 27. The remaining rods were sampled on day 42 (sample point W). Planktonic indigenous gut microbiota (periods A–C, vessels 2 and 3), *C. difficile* total viable counts, spores and cytotoxin (periods B and C, vessels 1–3) were monitored during the experiment. The experiment was performed twice to assess reproducibility.

### Enumeration of gut microbiota and *C. difficile* total viable counts, spores and cytotoxin

Planktonic and biofilm-associated gut microbiota populations cultured were: total facultative anaerobes, total anaerobes (facultative + obligate), lactose fermenting *Enterobacteriaceae*, enterococci, lactobacilli, bifidobacteria, total *Clostridium* spp., *Bacteroides fragilis* group, *C. difficile* total viable counts (vegetative *C. difficile* + spores) and *C. difficile* spore viable counts. *C. difficile* toxin production was monitored using a Vero cell cytotoxicity assay as described previously [18]. Planktonic cultures were serially diluted in pre-reduced peptone water to 10^−7^. Twenty microliters of four appropriate dilutions were inoculated to quarter plates of each culture medium in triplicate and incubated anaerobically (obligate anaerobes) or aerobically (facultative anaerobes) at 37°C for 48 hours. Colonies were identified to genus level on the basis of colony morphology, Gram reaction and microscopic appearance on selective and non selective agars (table 1).

**Table 1:** Culture medium used for the isolation and enumeration of indigenous gut microbiota and *C. difficile* (all agar bases are supplied by Oxoid, with the exception of CCEYL and FAA supplied by LabM, and made according to the manufacturer's instructions).

Medium	Target species
Fastidious anaerobe agar (FAA) supplemented with 5% horse blood	Total anaerobes and total clostridia
Bacteroides Bile Aesculin agar (BBE) supplemented with 5 mg/L haemin, 10 µL/L vitamin K, 7.5 mg/L vancomycin, 1 mg/L penicillin G, 75 mg/L kanamycin, 10 mg/L colisitin	*B. fragilis* group.
LAMVAB agar: 52.5 mg/L MRS broth, 20 mg/L agar technical supplemented with 0.5 g/L cysteine HCl, 20 mg/L vancomycin, adjusted to pH 5	*Lactobacillus* spp.
Beerens agar: 42.5 mg/L Columbia agar, 5 mg/L agar technical supplemented with 5 mg/L glucose, 0.5 g/L cysteine HCl, 5 mL propionic acid, adjusted to pH5	*Bifidobacterium* spp.
Nutrient agar	Total facultative anaerobes
MacConkey agar	Lactose-fermenting *Enterobacteriaceae*
Kanamycin Aesculin Azide agar supplemented with 10 mg/L nalidixic acid, 10 mg/L aztreonam, 20 mg/L kanamycin	*Enterococcus* spp.
Alcohol shock followed by Brazier's CCEY agar supplemented with 2% lysed horse blood, 5 mg/L lysozyme, 256 mg/L cycloserine, 8 mg/L cefoxitin	*C. difficile* spores
Brazier's CCEYL agar as described above supplemented with 2 mg/L moxifloxacin	*C. difficile* total viable counts

Biofilm –associated bacterial populations were enumerated following careful removal of rods from the biofilm model. Rods were transferred into 5 mL pre-reduced saline, thoroughly vortexed for ∼2 mins and the rod discarded. Re-suspended biofilm was centrifuged at 4000 rpm for 10 mins, the supernatant was then removed and re-suspended in 2 mL pre-reduced saline. A 500 µL aliquot was centrifuged and stored at 4°C for cytotoxin assay as previously described [18]. The remaining biofilm suspension was enumerated as described above and units were reported as log_10_ cfu/g of biofilm.

## Results

### Models A1 and A2

All rods facilitated the growth of biofilm within the single stage gut model. The macroscopic biofilm mass differed amongst rods with total pellet weight ranging from 0.034 to 0.122 g with an average (±SE) weight of 0.068±0.007 g in model A1, and 0.021 to 0.082 g with an average (±SE) weight of 0.046±0.004 g in model A2. In general there was no correlation between biofilm biomass and rod position, or rod surface topography (smooth or roughened/ground) between different experiments. This indicates that, although there are differences in biofilm biomass, these are not due to microenvironments within the vessel).

### Planktonic gut microbiota

The majority of indigenous gut microbiota in both models A1 and A2 established a steady population, with the exception of *B. fragilis* group, which declined to below the limit of detection in model A1 and was only sporadically detected at low levels (∼2–5 log_10_ cfu/mL) during period A in model A2 (data not shown).

### Sessile gut microbiota

**Figure 2:**
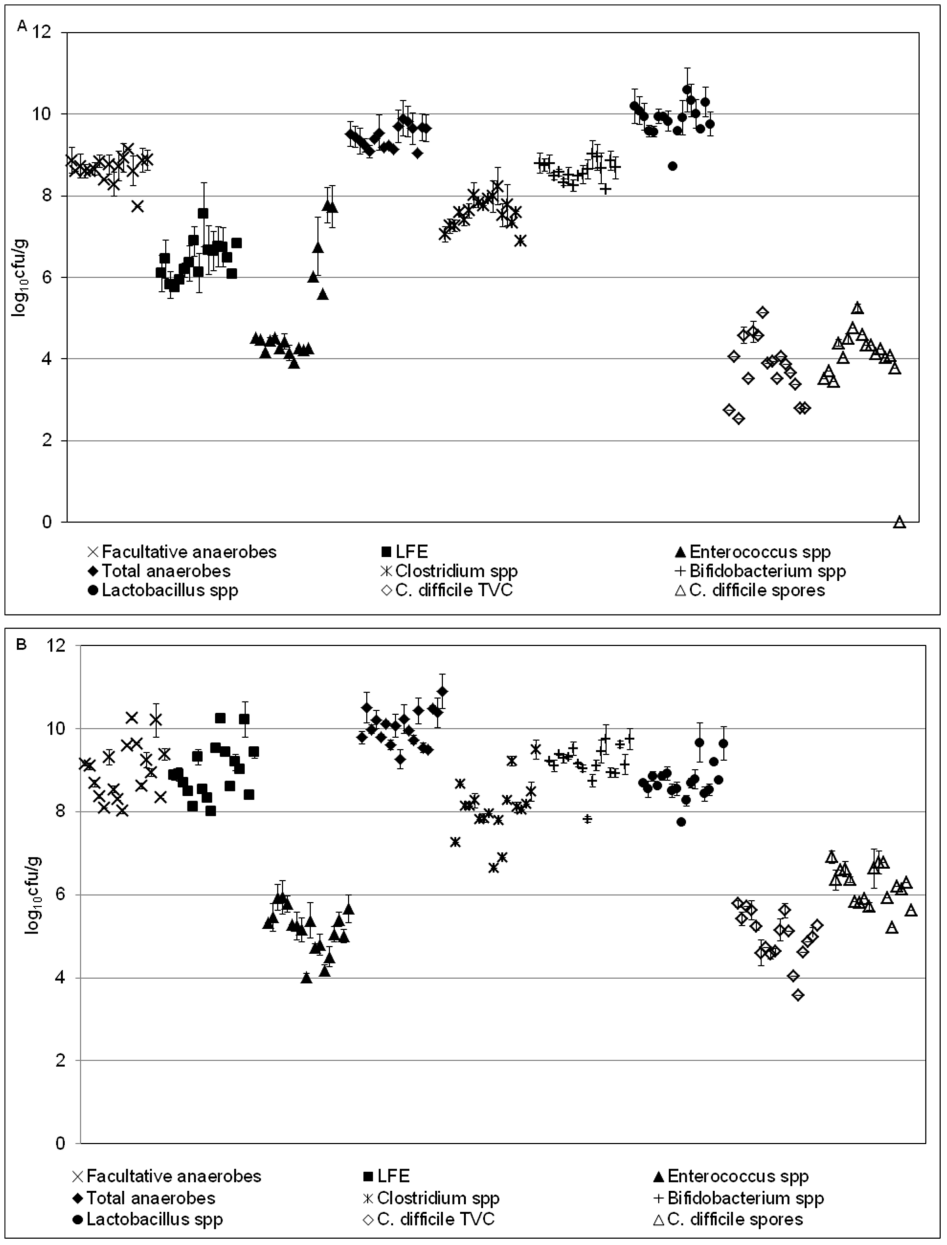
Sessile bacterial populations in models A1 and A2. Mean (±SE) rod-associated populations (log_10_ cfu/g) of indigenous gut microbiota within the single stage biofilm gut model (a-model A1; b-model A2). Each point represents the populations present on a single rod. *B. fragilis* group – below limit of detection (∼3 log_10_ cfu/g). LFE – Lactose-fermenting *Enterobacteriaceae*.

Sessile communities of all enumerated bacterial groups, except *B. fragilis* group were present on the glass rods (figure 2). Within model A1, *Lactobacillus* spp. dominated biofilm populations (mean 9.88 log_10_ cfu/g), whilst *Bifidobacterium* spp. (mean 9.18 log_10_ cfu/g) dominated sessile populations within model A2. *Enterococcus* spp. (mean 5.03 and 5.16 log_10_ cfu/g in model A1 and A2, respectively) was the least abundant bacterial group in both models. The relative proportions of each bacterial group were similar in planktonic and sessile form, although planktonic population counts were generally greater than their sessile counterparts.

Variation between sessile bacterial populations across all rods in each experiment (model A1 and A2) was minimal (table 2), with standard errors (SE) of variation of most bacterial groups ≤0.18 log_10_ cfu/g. *Enterococcus* spp. demonstrated greatest variation within model A1 (0.3 log_10_ cfu/g). Within model A1, *Enterococcus* spp. population variation between rods 1–12 (SE ±0.05 log_10_ cfu/g) was minimal and comparable to that of other bacterial groups. However, increased variation was evident on rods 13–17 (SE ±0.57 log_10_ cfu/g), a trend not observed for other bacterial groups on these particular rods. Variation of this bacterial group in model A2 was comparable to that of other bacterial groups.

**Table 2:** Mean and standard error values (log_10_ cfu/g) of sessile and planktonic populations of indigenous gut microbiota and *C. difficile* on rods 1–17 in models A1 and A2. TVC – total viable counts; NR – no result, LOD – limit of detection.

Bacterial group	Mean sessile (log_10_ cfu/g)		Standard error of sessile counts (log_10_ cfu/g)		Mean planktonic (log_10_ cfu/g)		
	Model A1	Model A2	Model A1	Model A2	Model A1	Model A2	
Facultative anaerobes	8.66	8.99	0.07	0.02	9.51	10.40	
Lactose-fermenting *Enterobacteriaceae*	6.42	8.96	0.11	0.15	6.62	10.39	
*Enterococcus* spp.	5.03	5.16	0.30	0.13	4.70	5.83	
Total anaerobes	9.44	10.03	0.06	0.10	10.92	11.35	
*Clostridium* spp.	7.59	8.07	0.09	0.16	9.23	NR	
*Bifidobacterium* spp.	8.61	9.18	0.06	0.10	10.81	10.09	
*B. fragilis* gp.	<LOD	<LOD	<LOD	<LOD	<LOD	<LOD	
*Lactobacillus* spp.	9.88	8.73	0.10	0.11	10.40	9.32	
*C. difficile* TVC	3.74	4.97	0.18	0.14	<LOD	<LOD	
*C. difficile* spores	4.19	6.20	0.11	0.11	3.45	3.20	

### Planktonic and sessile C. difficile

*C. difficile* spores were instilled into the vessel on day 31 and remained in spore form for the duration of the experiment in both models. Planktonic populations declined to the limit of detection by the final day of the experiment (data not shown). *C. difficile* were detected in spore form within rod-associated biofilms on the final day of the experiment, with sessile populations up to 3 log_10_ cfu/g greater than their planktonic counterparts.

### Models B1 and B2

#### Planktonic gut microbiota

Vessel 3 was the only biofilm vessel, and so is of most relevance for validation against planktonic populations; therefore, only indigenous gut microbiota results from vessel 3 are reported here. Planktonic indigenous gut microbiota rapidly established a stable population, and remained predominantly steady for the duration of the experiment (data not shown). Instillation of *C. difficile* spores (day 21, period B) did not substantially affect microbiota populations.

#### Sessile gut microbiota

**Figure 3:**
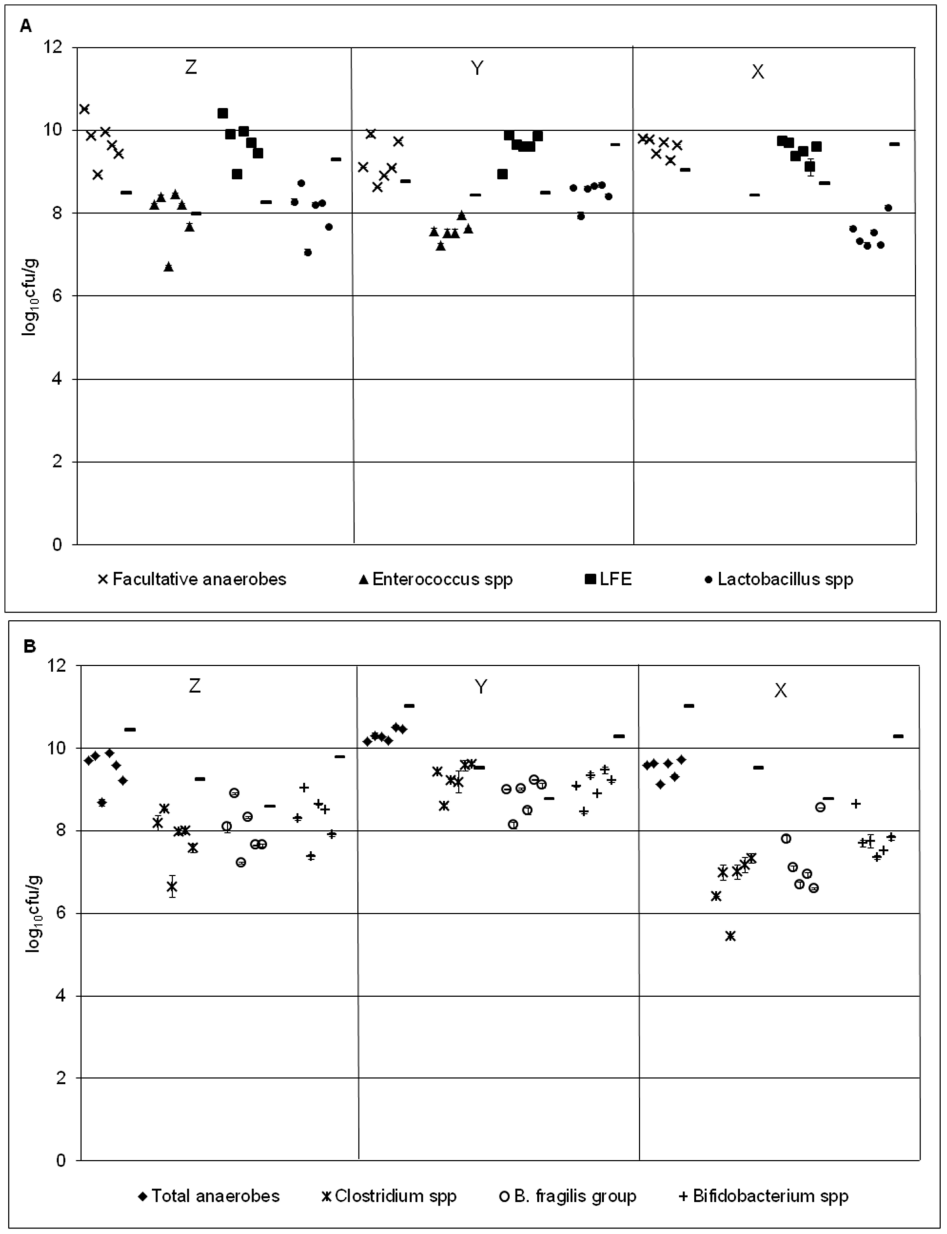
Bacterial populations in model B1. Mean (± SE) rod-associated populations (log_10_ cfu/g) of (a) facultative (b) obligate anaerobes within the triple stage biofilm gut model (model B1) at time points Z- X. Planktonic populations of each bacterial group (log_10_ cfu/g) at each time point are represented by a line. LFE – lactose-fermenting *Enterobacteriaceae*.

**Figure 4:**
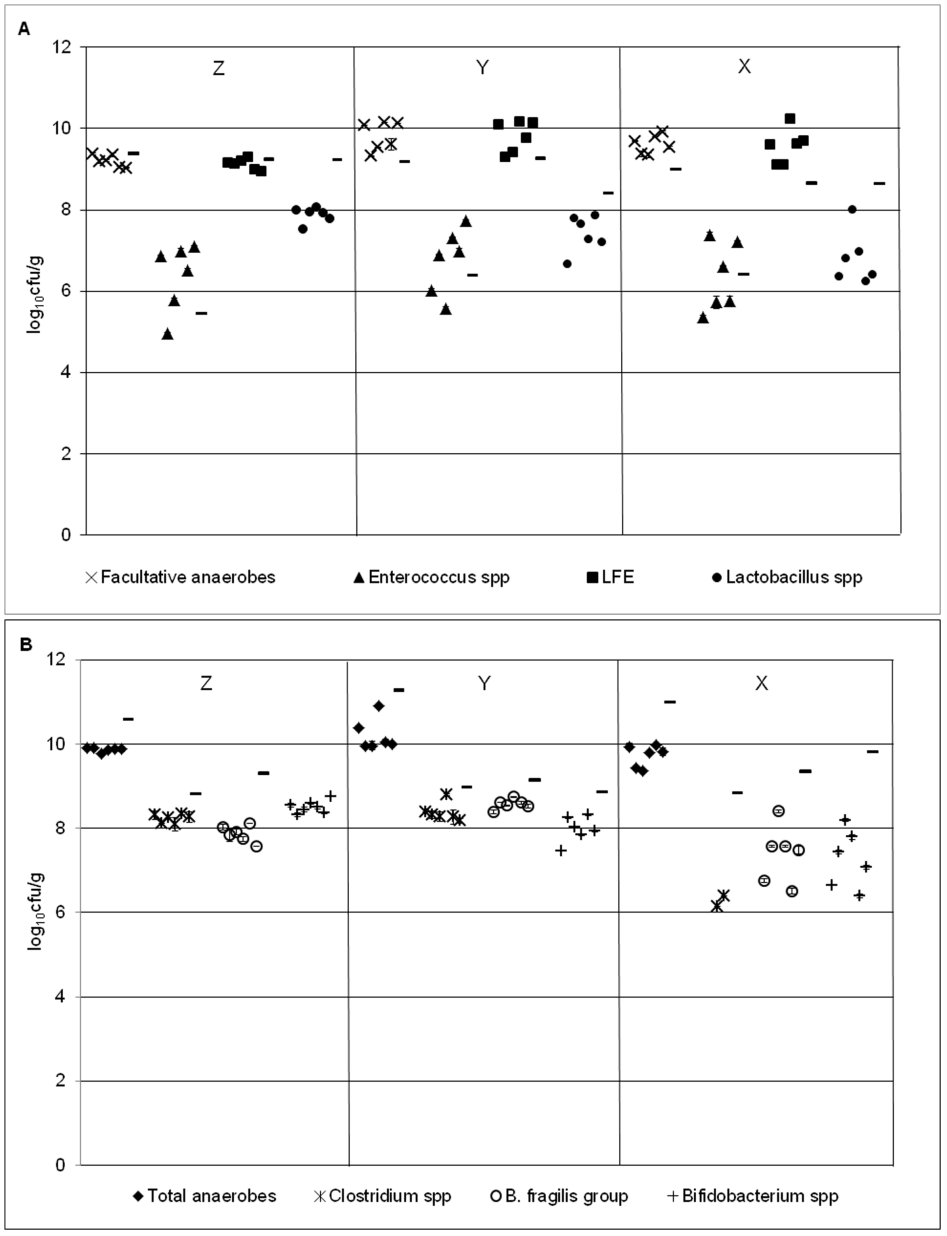
Bacterial populations in model B2. Mean (± SE) rod-associated populations (log_10_ cfu/g) of (a) facultative (b) obligate anaerobes within the triple stage biofilm gut model (model B2) at time points Z- X. Planktonic populations of each bacterial group (log_10_ cfu/g) at each time point are represented by a line. LFE – lactose-fermenting *Enterobacteriaceae*.

Bacterial group populations across the 6 rods sampled at each time point displayed minimal variation, with standard errors of ≤0.35 log_10_ cfu/g at all time points (table 3). Biofilm communities were dominated by lactose-fermenting *Enterobacteriaceae*, with *Enterococcus* spp. the least numerous bacterial group for all time points (figures 3 & 4). Rod-associated populations for each bacterial group remained largely steady across the 3 time points, although *Clostridium* spp., *B. fragilis* group and *Bifidobacterium* spp. populations reduced modestly at time point X (∼1–2 log_10_ cfu/g decline). Variations (SE) of sessile populations at time point X were generally more pronounced compared with previous time points.

**Table 3:** Mean (log_10_ cfu/g) and standard error (SE) of rod-associated populations of indigenous gut microbiota at sample points Z, Y and X in the triple stage biofilm gut model B1 and B2. NR – no result.

Bacterial group	Sessile mean ±SE Sample point Z		Sessile mean ± SE Sample point Y		Sessile mean ± SE Sample point X	
	B1	B2	B1	B2	B1	B2
Facultative anaerobes	9.71 (±0.07)	9.22 (±0.06)	9.22 (±0.20)	9.80 (±0.15)	9.60 (±0.08)	9.61 (±0.09)
Lactose-fermenting *Enterobacteriaceae*	9.70 (±0.11)	9.10 (±0.05)	9.57 (±0.14)	9.80 (±0.15)	9.49 (±0.09)	9.54 (±0.17)
*Enterococcus* spp.	7.94 (±0.30)	6.36 (±0.34)	7.57 (±0.09)	6.75 (±0.33)	NR	6.34 (±0.35)
Total anaerobes	9.48 (±0.06)	9.87 (±0.02)	10.32 (±.006)	10.21 (±0.15)	9.51 (±0.10)	9.72 (±0.11)
*Clostridium* spp.	7.82 (±0.09)	8.24 (±0.09)	9.27 (±0.15)	8.37 (±0.09)	6.72 (±0.28)	6.24 (±0.07)
*Bifidobacterium* spp.	8.29 (±0.06)	8.46 (±0.04)	9.07 (±0.15)	7.97 (±0.13)	7.79 (±0.18)	7.26 (±0.30)
*B. fragilis* gp.	7.97 (±0.24)	7.87 (±0.08)	8.82 (±0.17)	8.58 (±0.05)	7.27 (±0.31)	7.38 (±0.28)
*Lactobacillus* spp.	8.03 ±0.10	7.87 ±0.08	8.49 ±0.12	7.42 ±0.18	7.52 ±0.14	6.81 ±0.26
*C. difficile* TVC	NR	NR	6.19 ±0.13	5.38 ±0.13	4.49 ±0.40	4.79 ±0.14
*C. difficile* spores	NR	NR	6.62 ±0.19	5.51 ±0.14	5.53 ±0.28	4.83 ±0.12

#### Planktonic C. difficile

*C. difficile* populations initially remained quiescent in all vessels of the gut model (data not shown). However, ∼5 days after *C. difficile* spore instillation, TVCs began to increase by ∼1–2 log_10_ cfu/mL relative to spore populations and maintained this level for the duration of the experiment. *C. difficile* spore populations decreased from peak levels of 4.5 log_10_ cfu/mL to ∼3 log_10_ cfu/mL by the end of the experiment. Cytotoxin was not detected throughout the experiment in model B1, but was observed (2RU) on the penultimate day of the experiment in model B2 (data not shown).

#### Sessile C. difficile

**Figure 5:**
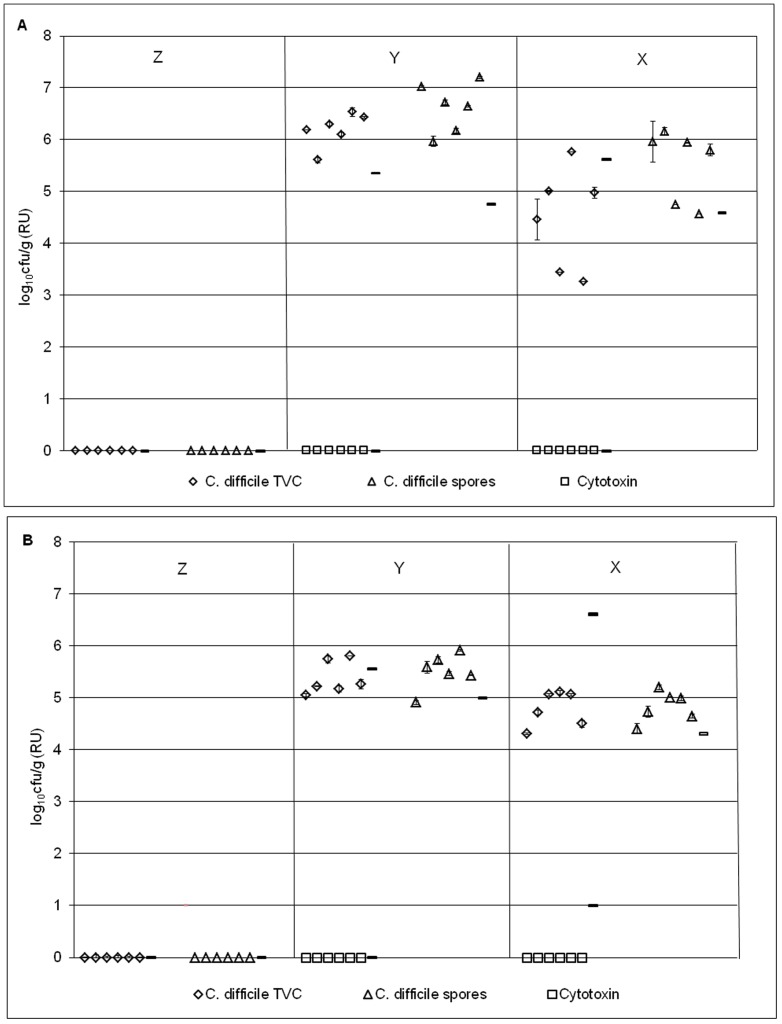
*C. difficile* populations in models B1 and B2. Mean (± SE) rod-associated populations (log_10_ cfu/g) of *C. difficile* within the triple stage biofilm gut model (model B1 – a; model B2 - b) at time points Z- X. Planktonic *C. difficile* populations (log_10_ cfu/g) at each time point are represented by a line.

Following *C. difficile* spore instillation (day 21; post time point Z), *C. difficile* populations existed solely as spores, with no indication of vegetative cell presence within all rod-associated biofilm structures at time points X and Y in models B1 and B2 (figures 5a and b). *C. difficile* spores were present within biofilm structures (mean TVCs 5.51–6.62 log_10_ cfu/g), 6 days after spore introduction into the model (time point Y, figures 5a and b). *C. difficile* populations existed in spore form only, with little population variation amongst rods (standard error: TVCs and spores 0.13–19 log_10_ cfu/g, table 3). On the final day of the experiment (day 46, time point X), mean sessile *C. difficile* spores had declined to 4.83 and 5.53 log_10_ cfu/g in models B1 and B2, respectively (time point X, figures 5a and b). Variation (SE) of *C. difficile* populations amongst rods at time point X was greater than other bacterial groups with TVC and spore standard errors of 0.12–0.4 log_10_ cfu/g.

## Discussion

A well established, validated *in vitro* human gut model of simulated *C. difficile* infection has been adapted to incorporate a rod system to facilitate the independent formation and analysis of 18 rod-associated multi-species biofilms, thus allowing longitudinal analysis of composition over weeks or months. In order to allow future work comparing biofilm composition at different time points, and under different conditions, the consistency of biofilm formation and composition across all rods, regardless of position within the vessel was validated. The validation process was initially carried out within a single stage model (i.e. the biofilm vessel only) and later in a triple stage system where vessel 3, representing the distal colon, was replaced with the biofilm vessel. These experiments were performed in duplicate and demonstrate the high level of reproducibility of the model. Due to the time and labour intensive nature of this model the practicality of numerous repeat experiments is limited. The measurement of planktonic populations within this model has previously been validated and repeated on numerous occasions, with high level of reproducibility observed [19]–[22]. Further work with the biofilm model would provide increased confidence in the reproducibility of sessile populations but were beyond the limits of this project.

The biofilm gut model provides a reproducible system in which to study the growth of mature, multi-species biofilm. Maximal biofilm formation was macroscopically evident at the liquid/gas interface on each rod, with a plaque-like biofilm growth on submerged areas. Attempts were made to ensure a homogenous environment for biofilm formation; however, some variation in biofilm biomass was evident across the 18 rods, although no relationship between rod position and biofilm biomass was evident. Microenvironments within the model were evident, with variations in fluid turbidity likely due to gas flow. Shear forces and turbidity levels within a fluid regulate biofilm formation, with turbid environments eliciting increased biofilm formation [23], potentially explaining the biomass variation observed within this study. Roughened and smooth glass rods were utilised in models A1/2. Despite reports of surface topography affecting biofilm formation [24], [25], no evidence of variation in biofilm mass or composition were evident in this study. Previous reports focus on the differences in early bacterial colonisation rates on different materials [25], [26], a parameter not investigated within this study. However, robustness of biofilm structures on roughened rods was greater than those on smooth glass rods.

The triple stage experimental design (models B1 and B2) facilitated the investigation of sessile communities at 3 distinct time points, with rod-associated biofilm biomass generally decreasing as the experiment progressed. The biofilm lifecycle is a dynamic process and variation in biofilm biomass identified at these distinct time points may be a result of individual rod-associated biofilms existing at difference stages. Biofilm detachment is regulated by factors such as pH, nutrient availability and sheer forces [23], [27], and therefore decreased mass may be due to communities reaching increased structural stability and optimal biomass at different stages in the experiment.

Despite vessel design alteration compared with the original gut model, planktonic indigenous gut microbiota and *C. difficile* populations within the biofilm vessel of all models (A1/2 and B1/2) closely reflected observations in previous gut model experiments [14], [28]. Slight variation (average, 0.7 log_10_ cfu/g; maximum 2.5 log_10_ cfu/g) in planktonic indigenous gut microbiota populations was evident between repeating experiments (models A1/2 and models B1/2), potentially caused by inter- and intra-bacterial variation in donor stools [4]. *B. fragilis* group populations were detected sporadically at low levels within the planktonic cultures of the single stage models, as observed previously within the gut model [29]. Detection of *Bacteroides* spp. within distinct regions of the colon of sudden death victims are variable [30]. Reasons behind these differences are unclear, but may be mediated by the reduced pH, increased substrate availability in vessel 1 (proximal) compared to vessel 3 (distal) and resulting unique fermentation profiles within different regions of the model [17].

Sessile bacterial group populations (log_10_ cfu/g) remained consistent across rods within each experiment and results from the single (models A1 and A2) and triple (model B1 and B2) stage models were reproducible. All bacterial groups isolated from the planktonic mode of growth were also present within the rod-associated biofilms, with the exception of *B. fragilis* group within the single stage models, correlating with the absence or low levels of this group within the planktonic culture.

In order to compare planktonic and sessile populations from a single time point, populations from both modes of growth were expressed as log_10_ cfu/g. In general, planktonic indigenous gut microbiota populations were higher than their sessile counterparts, as observed by Macfarlane *et al* [6], but not Probert and Gibson [31]. The weight of the pellet from a 1 mL suspension of planktonic culture fluid or re-suspended biofilm was used to determine the cfu per gram of biomass. Planktonic cultures are likely to contain a high proportion of bacteria within this biomass, whilst biofilm is comprised of a large amount of extracellular material in addition to the bacterial mass, possibly explaining this observation [32]. Sessile facultative anaerobic populations, particularly *Enterococcus* spp. were generally higher than planktonic populations. The lower ratio of anaerobes to aerobes at the gut mucosal surface compared with the lumen has previously been observed *in vivo* [4] and *in vitro* [6]. An increased level of variation in *Enterococcus* spp. populations relative to other bacterial groups was observed within these experiments, particularly model A1. Within model A1, green pigmentation, presumptively identified as pseudomonal growth was observed in biofilms on rods associated within increased *Enterococcus* spp. variation (rods 13–17, model A1). The association of *P. aeruginosa* with *E. faecalis* has previously been noted [33], potentially implying a symbiotic relationship between the two organisms. The presence of *P. aeruginosa* may lead to recruitment of increased levels of enterococci species into biofilm structures.

Within the original gut model an internal control period was designed to demonstrate the quiescent nature of *C. difficile* spores in the absence of antimicrobial intervention. During this period within models B1 and B2, planktonic *C. difficile* TVCs increased relative to spore levels, demonstrating the presence of low levels (∼1–2 log_10_ cfu/mL) of planktonic *C. difficile* vegetative/active cells within the model. Whilst germination of *C. difficile* spores appeared evident, proliferation of vegetative cells remained restricted, particularly in model B1. Persistence of planktonic *C. difficile* spore populations (i.e. washout absence), whilst unusual, has previously been observed within the original gut model [34], [35]. Whilst the presence of increased TVCs compared with spores was evident within planktonic *C. difficile* communities, there was no evidence of this trait within biofilm populations. Biofilm formation and sporulation pathways are interconnected and differentially regulated by the master regulator Spo0A in *Bacillus. subtilis* [36]. *C. difficile* possesses a Spo0A orthologue with 56% amino acid homology to that of *B. subtilis*. This orthologue plays a key role in *C. difficile* sporulation and biofilm formation [37], [38]. The *C. difficile* biofilm lifecycle is a complex process, which remains to be fully elucidated. Sessile bacteria possess distinct metabolic and phenotypic characteristics from their planktonic counterparts, including reduced growth rate. The recruitment of quiescent spores into a dormancy-inducing biofilm structure enhances the likelihood of spores remaining inactive. *C. difficile* spores germinate in response to external stimuli such as pH, heat and nutrients such as glycine and taurocholate [39]–[41]. Conditions within planktonic fluid of the human gut model may vary markedly from the protected environment of biofilms, potentially eliciting the diverse behaviour of *C. difficile* between the two phases. Results presented here demonstrate the planktonic and biofilm modes of growth of *C. difficile* PCR ribotype 027 only. Despite reduction in the prevalence of this ribotype within the UK, [42] it is still clinically relevant in many parts of the world and is associated with poor outcomes[43], [44]. Inter-strain spore coat heterogeneity is thought to exist within *C. difficile*, and may confer a greater adherence capability for certain strains. The further use of the biofilm gut model to investigate the association of different PCR ribotypes to biofilm structures may provide useful additional information on inter-strain heterogeneity and any potential clinical relevance.

The preferential persistence of *C. difficile* spores in the biofilm phase described here offers both a reinforcement of the need to examine planktonic and sessile populations and a tantalising suggestion that biofilms could play a key role in CDI recrudescence. Therapeutic options that can address *C. difficile* spore persistence, notably in biofilms, may have clinical advantage, for example in terms of reduced propensity for disease relapse.

The biofilm chemostat gut model has been validated for reproducible and consistent formation of mixed species intestinal biofilms. This model can be utilised for the analysis of sessile mixed species communities longitudinally, potentially providing further information on the persistence of *C. difficile* spores within biofilms and the role this may play in recurrent CDI. In particular, this could offer new insights into the efficacy of novel therapeutics for CDI.
